# Laparoscopic versus open surgery for pheochromocytoma: a meta-analysis

**DOI:** 10.1186/s12893-020-00824-6

**Published:** 2020-07-25

**Authors:** Sheng-Qiang Fu, Si-Yuan Wang, Qiang Chen, Yu-Tang Liu, Zhi-Long Li, Ting Sun

**Affiliations:** Department of Urology, the First Affiliated Hospital of Nanchang University, Nanchang, 330000 Jiangxi Province China

**Keywords:** Laparoscopic, Open, Pheochromocytoma

## Abstract

**Background:**

Surgical resection is the main treatment for pheochromocytoma (PHEO). Although open surgery (OS) has been shown to be safe and feasible, the safety and efficacy of laparoscopic surgery (LS) for PHEO remain controversial due to the uncertain effects of pneumoperitoneum on haemodynamics and the complexity of the tumour itself. This study was performed to compare the treatment outcomes of OS with those of LS for patients with PHEO.

**Methods:**

A systematic search through November 11, 2019, was conducted. All studies comparing outcomes of LS and OS for PHEO were included according to eligibility criteria. This meta-analysis was conducted using Review Manager Software, version 5.3, and STATA software, version 12.0. The quality of the included studies was assessed using the Newcastle-Ottawa scale.

**Results:**

Fourteen studies involving 626 patients were included in this meta-analysis. LS was associated with lower rates of intraoperative haemodynamic instability (IHD) [odds ratio (OR) = 0.61, 95% CI: 0.37 to 1.00, *P* = 0.05], less intraoperative blood loss [weighted mean difference (WMD) = − 115.27 ml, 95% confidence interval (CI): − 128.54 to − 101.99, *P* < 0.00001], lower blood transfusion rates [OR = 0.33, 95% CI: 0.21 to 0.52, *P* < 0.00001], earlier ambulation (WMD = − 1.57 d, 95% CI: − 1.97 to − 1.16, *P* < 0.00001) and food intake (WMD = − 0.98 d, 95% CI: − 1.36 to − 0.59, *P* < 0.00001), shorter drainage tube indwelling time (WMD = − 0.51 d, 95% CI: − 0.96 to − 0.07, *P* = 0.02) and postoperative stay (WMD = − 3.17 d, 95% CI: − 4.76 to − 1.58, *P* < 0.0001), and lower overall complication rates (OR = 0.56, 95% CI: 0.35 to 0.88, *P* = 0.01). However, no significant differences in operative time, postoperative blood pressure control, rates of severe complications, postoperative hypotension or cardiovascular disease (CVD) were found between the two groups.

**Conclusions:**

LS is safe and effective for PHEO resection. Compared with OS, LS caused less IHD, providing an equal chance to cure hypertension while also yielding a faster and better postoperative recovery.

## Background

Since Gagner and colleagues first reported laparoscopic adrenalectomy in 1992 [1], laparoscopic surgery (LS) has been gaining popularity around the world. Currently, LS is widely accepted as a well-established procedure for the removal of benign adrenal neoplastic diseases, such as Cushing’s syndrome, primary aldosteronism and nonfunctional adrenal tumours, because it is considered safe, effective, and less invasive than conventional open surgery (OS) [2, 3]. Pheochromocytoma (PHEO) is a neuroendocrine tumour originating from the adrenal medulla, and surgical removal is the main treatment [4]. However, the removal of PHEO is a huge challenge for surgeons. In addition to the risks and complications associated with general adrenal surgery, this procedure can induce a surge of catecholamines, which can further lead to dramatic haemodynamic fluctuations and even perioperative mortality [5]. Conventional OS for PHEO has yielded great results in the past, but the procedure is traumatic and involves a large surgical incision. Recently, an increasing number of studies have suggested that LS is a safe and feasible procedure for PHEO and might become a more optimal approach than OS [6–8]. Indeed, LS was found in many studies to be superior to OS in terms of treatment outcomes. Nonetheless, most of these studies involved an insufficient number of enrolled patients and a single institution, rendering the advantages of LS in these studies less convincing. Therefore, this meta-analysis was conducted to compare treatment outcomes of LS and OS for PHEO based on current retrievable studies. This study complies with PICOS principles [9].

## Methods

### Literature search

A systematic search in electronic databases (PubMed, Web of Science, EMBASE, and Cochrane Library) was implemented to identify eligible studies comparing LS and OS for PHEO published through November 11, 2019. The following search terms were used: “laparoscop*”, “minimally invasive”, “pheochromocytoma*”, “phaeochromocytoma*”, “chromaffinoma*”, and “chromaffin tumour*”. The wildcard character and combined Boolean operators “OR” or “AND” or “NOT” Title/Abstract were applied to achieve an efficient literature search. The language of the literature was limited to English. References in related literature were manually searched for potential studies. All studies that met the inclusion criteria were reviewed for further data extraction. This study was conducted following the Preferred Reporting Items for Systemic Reviews and Meta-Analysis (PRISMA) statement [10], and the work is reported in adherence to the Assessing the Methodological Quality of Systematic Reviews (AMSTAR) guidelines [11].

### Inclusion and exclusion criteria

The titles and abstracts of the literature identified were scanned by two authors (SQF and SYW) independently. After carefully reviewing the full texts, relevant articles were further identified. When a dispute arose, a third party intervened to achieve a resolution through joint discussion. Studies were included in the present meta-analysis when they conformed to the following inclusion criteria: (1) comparative studies, comparing the treatment outcomes of LS with OS for PHEO; (2) accepted or published articles, with full texts available; and (3) each article reported two or more treatment outcomes (see below). Studies were excluded if the following exclusion criteria were met: (1) PHEO reported in the study was bilateral or extra-adrenal or with distant metastases; and (2) guidelines, reviews, conference abstracts, letters, case reports, or other types of literature for which data cannot be extracted.

### Data extraction and quality assessment

SQF and SYW reviewed each article in a list and extracted data into a table that collated the data prepared in advance. When disputes arose during data extraction, a consensus was reached through discussion with a third party. Data concerning the following were extracted: (1) general characteristics, including first author, publication year, country, study type, sex and age of patients, and body mass index; and oncology characteristics, including tumour size, tumour laterality, postoperative pathology, perioperative mortality, and postoperative recurrence; (2) intraoperative outcomes, including intraoperative haemodynamic instability (IHD), operative time, intraoperative blood loss and the number of blood transfusions; IHD was defined as intra-operative systolic blood pressure > 200 mmHg or mean arterial pressure < 60 mmHg requiring drugs or blood transfusions to main normal blood pressure intra-operatively [12]; (3) postoperative outcomes, including time to ambulation and diet, indwelling time of drainage tube, postoperative hospital stay, overall rates and severity (Clavien-Dindo grade ≥ II) of postoperative complications, rates of postoperative hypotension and cardiovascular disease (CVD) morbidity, and postoperative blood pressure control during follow-up. CVD morbidity was defined as complications related to the cardiovascular system, such as postoperative hypotension requiring drugs or blood transfusion, myocardial ischaemia, arrhythmia, and pulmonary embolism/cerebral infarction/deep vein thrombosis [13]. Disputes encountered during data extraction were resolved through discussion with third parties. Recovery of a patient’s bowel function was predicted by the onset of feeding. The severity of complications was assessed by the sum number of relevant complications reported in each study. The Newcastle-Ottawa scale (NOS) was used to assess the methodological quality of the studies [14]. QC and YTL evaluated the quality of each included study independently. When a dispute was encountered, resolution through joint discussion was achieved with a third party. The scale ranged from 0 to 9 stars, and studies with a score ≥ six stars were deemed to be of high quality [15].

### Statistical analysis

All meta-analyses were conducted by Review Manager Version 5.2 software (The Cochrane Collaboration, Oxford, UK). Odds ratios (ORs) with 95% confidence intervals (CIs) were employed for analysis of dichotomous variables; the weighted mean difference (WMD) with 95% CIs was used for continuous variables. Continuous variables are presented as medians and ranges, and the mean and standard deviation (SD) of these variables were estimated as reported by Hozo et al. [16]. Heterogeneity among the studies was assessed by the I^2^ statistic [17], whereby the random effects model was adopted for I^2^ > 50% and the fixed effects model for I^2^ was < 50% [17]. Funnel plots and Begg’s test (STATA software, version 12.0) were used to assess publication bias. A *p* value < 0.05 indicated a statistically significant difference.

## Results

### Search results and study characteristics

**Fig. 1:**
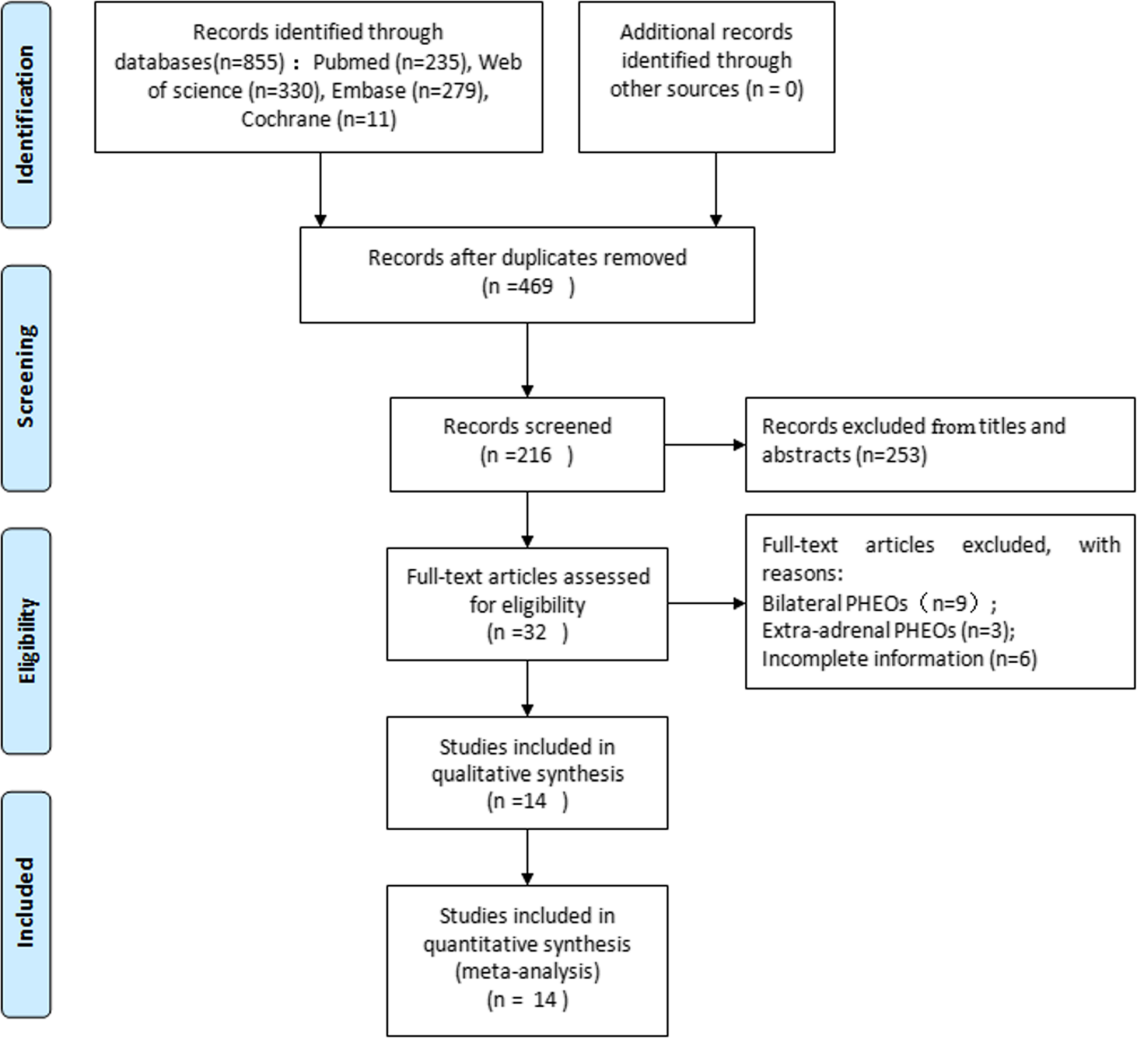
Flow chart of the literature retrieval strategies. PHEO: pheochromocytoma

Initially, 855 potential studies were obtained by searching PubMed, Web of Science, EMBASE, and Cochrane Library. After removing duplicates, the remaining 469 relevant studies were further screened, and 253 were eliminated by scanning the titles and abstracts; full text review was performed on 32 articles. After reviewing the full text, 14 studies that met the criteria were included in the meta-analysis [6–8, 13, 18–27]. A flow chart of the literature retrieval summary is depicted in Fig. 1. The general characteristics of each study and the quality of the included non-randomized studies evaluated by the NOS are listed in Table 1. The oncological characteristics of the included studies are shown in Table 2.

**Table 1:** General characteristics of the included studies

Author-year	Country	Study type	Gender (M/F)		Age (yr)^**c**^		BMI (kg/m^**2**^)^**c**^		Quality score^**d**^
			LS	OS	LS	OS	LS	OS	
**Tiberio-2008**	**Italy**	**RCT**	**7/6**	**7/2**	**51 (37–74)**	**51 (34–61)**	**NR**	**NR**	**/**
**Inabnet-2000**	**France**	**RS**	**3/8**	**4/7**	**51.4± 10.0**	**45.7±14.0**	**NR**	**NR**	**8**
**Kim-2004**	**Korea**	**RS**	**8/7**	**5/4**	**45.2 (30-58)**	**43.3 (23-66)**	**24.2±1.8**^**a**^	**24±1.2**^**a**^	**7**
**Zhu-2019**	**China**	**RS**	**24/22**	**28/18**	**48.15±12.68**	**45.17±10.29**	**21.62±2.70**	**21.67±2.73**	**8**
**Bai-2019**	**China**	**RS**	**27/37**	**31/33**	**51.9 ± 13.3**	**51.7 ± 13.4**	**23.4(20.4-25.1)**^**b**^	**23.1(20.2-26.1)**^**b**^	**8**
**Sprung-2000**	**America**	**RS**	**5/9**	**8/12**	**57 ± 15**	**46 ± 17**	**NR**	**NR**	**7**
**Tanaka-2000**	**Japan**	**RS**	**NR**	**NR**	**39.5 (16-50)**	**38 (15-63)**	**NR**	**NR**	**7**
**Lang-2008**	**China**	**RS**	**30/23**	**26/24**	**36.2±12.5**	**35.4±14.1**	**23.3±1.3**	**23.5±1.4**	**8**
**Edwin-2001**	**Russia**	**RS**	**NR**	**NR**	**52 (31–65)**	**45 (24–65)**	**NR**	**NR**	**7**
**Wang-2011**	**China**	**RS**	**11/15**	**14/9**	**42.4 ± 11.7**	**37.5 ± 16.3**	**23.5 ± 2.96**	**23.0 ± 3.46**	**7**
**Davies-2004**	**Victoria**	**RS**	**6/6**	**7/5**	**54±15**	**52±22**	**NR**	**NR**	**8**
**Kazaryan-2004**	**Russia**	**RS**	**5/4**	**9/13**	**48 ± 12**	**44 ± 12**	**NR**	**NR**	**8**
**Song-2012**	**China**	**RS**	**13/10**	**16/9**	**32-67**	**28-62**	**NR**	**NR**	**7**
**Barband-2008**	**Iran**	**RS**	**5/5**	**6/4**	**36±13.7**	**42.3±15.2**	**24.7±2.3**	**26.2±1.7**	**8**

*BMI* Body mass index, *F* Female, *M* Male, *LS* Laparoscopic surgery, *OS* Open surgery, *NR* Not reported, *RCT* Randomized controlled trials, *RS* Retrospective study

^a^The data are expressed as the mean ± standard error

^b^Non-normal continuous variables are expressed as the median (interquartile range)

^c^The data are expressed as the mean ± SD or medians (range)

^d^According to the Newcastle-Ottawa scale classification

**Table 2:** Oncology outcomes of the included studies

Author year	TS (cm)	TL (R/L)	PP (Pn/Tn)	PM (n)	PR (n)	FT (Month)
Tiberio-2008^a^	4.0 (2.2-6.0)	8/5	13/13	0	0	18
Tiberio-2008^b^	4.1 (2.5-6.0)	5/4	9/9	0	0	18
Inabnet-2000^a^	4.1±1.2	8/3	11/11	0	0	37 (26-51)
Inabnet-2000^b^	4.6±1.2	3/8	11/11	0	0	52 (27-72)
Kim-2004^a^	5.2 ± 2.0	9/6	15/15	0	NR	22
Kim-2004^b^	6.4 ± 2.6	5/4	9/9	0	NR	36
Zhu-2019^a^	7.76± 2.02	29/15	NR	0	NR	40.9 (9.5, 102.5)
Zhu-2019^b^	7.92± 1.98	28/16	NR	0	NR	70.8 (4.0, 117.5)
Bai-2019^a^	7.8 (7.0-8.6)	34/30	NR	0	3	36
Bai-2019^b^	8.2 (7.0-10.0)	30/34	NR	2	1	65
Sprung-2000^a^	NR	NR	14/14	0	NR	NR
Sprung-2000^b^	NR	NR	20/20	0	NR	NR
Tanaka-2000^a^	3.5 (3.0-5.0)	NR	NR	0	NR	NR
Tanaka-2000^b^	4.1 (2.3-6.2)	2/4	NR	0	NR	NR
Lang-2008^a^	4.5 ± 2.0	25/28	53/53	0	0	5-36
Lang-2008^b^	4.9 ± 2.6	27/23	53/53	0	0	5-36
Edwin-2001^a^	6.0 (3.0-11.0)	1/5	6/6	0	NR	NR
Edwin-2001^b^	6.0 (2.7–8.0)	4/4	8/8	0	NR	NR
Wang-2011^a^	4.06 ± 1.47	10/16	NR	0	0	25 (20-40)
Wang-2011^b^	5.61 ± 2.74	13/10	NR	0	0	25 (20-40)
Davies-2004^a^	NR	3/9	11/11	0	NR	NR
Davies-2004^b^	NR	5/7	12/12	0	NR	NR
Kazaryan-2004^a^	6.4 ± 2.6	3/6	9/9	0	NR	NR
Kazaryan-2004^b^	5.6 ± 1.9	10/12	22/22	0	NR	NR
Song-2012^a^	1.5-4.0	11/12	23/23	0	0	6-48
Song-2012^b^	2.0-5.5	11/14	25/25	0	0	6-48
Barband-2008^a^	3.8±0.77	6/4	NR	0	NR	NR
Barband-2008^b^	5.8±3.1	NR	NR	0	NR	NR

*FT* Follow-up time, *L* Left, *NR* Not reported, *PM* Perioperative mortality, *Pn* The number of pheochromocytomas, *PR* Postoperative recurrence, *PP* Postoperative pathology, *R* Right, *TL* Tumor laterality, *Tn* The number of patients, *TS* Tumor size

^a^Patients in this group underwent laparoscopic surgery

^b^Patients in this group underwent open surgery

### Intraoperative outcomes

**Fig. 2:**
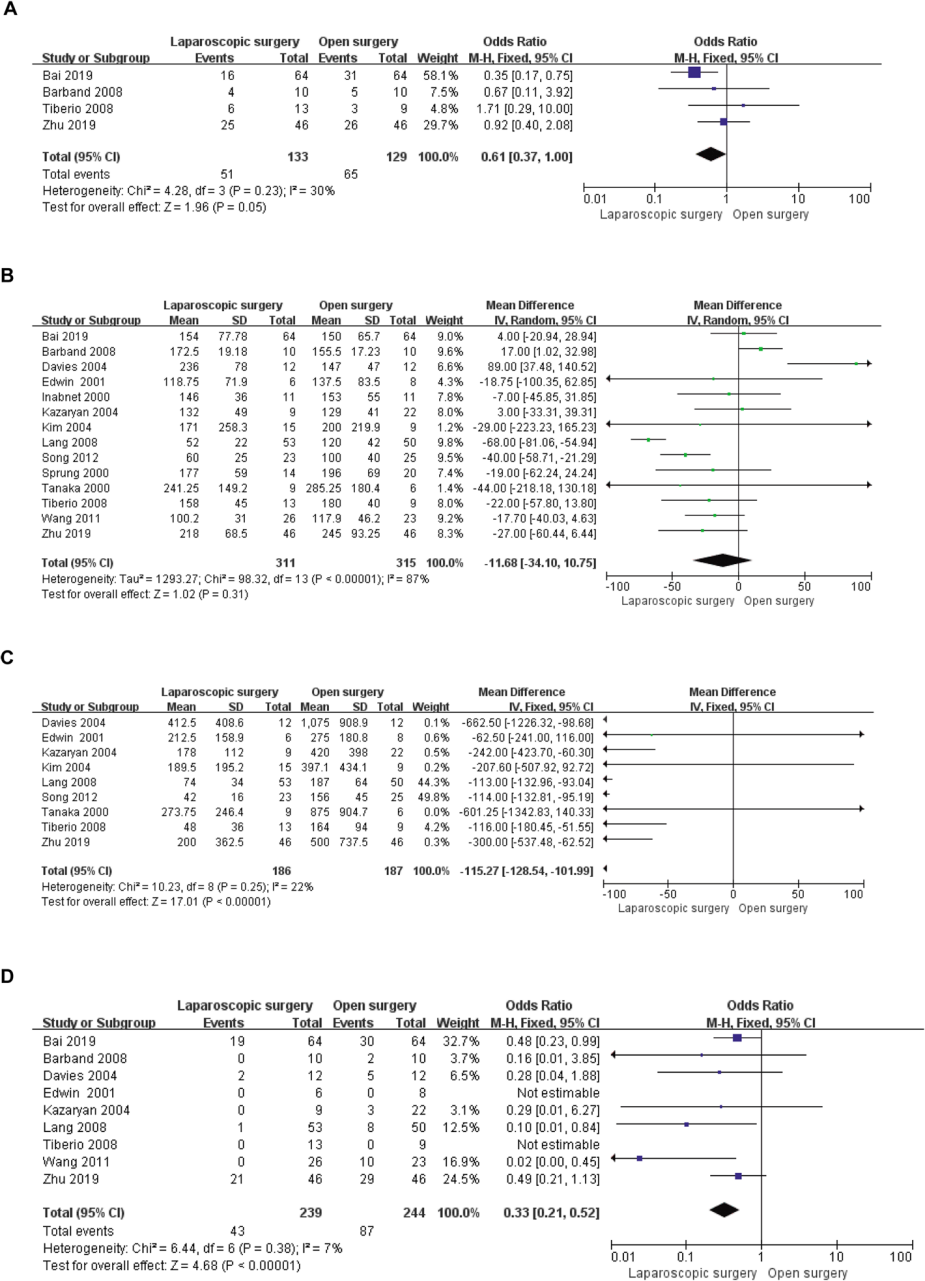
Forest plots of the meta-analysis for intraoperative outcomes. **a**: The number of IHD cases was compared between the LS and OS groups. **b**: Operative time was compared between the LS and OS groups. **c**: Intraoperative blood loss was compared between the LS and OS groups. **d**: The number of intraoperative blood transfusions was compared between the LS and OS groups. IHD: Intraoperative haemodynamic instability; LS: Laparoscopic surgery; OS: Open surgery; CI: Confidence interval; SD: Standard deviation

Intraoperatively, outcome indicators in the present study included IHD, operation time, intraoperative blood loss and number of intraoperative blood transfusions. Compared to the OS group, fewer patients in the LS group presented haemodynamic instability (OR = 0.61, 95% CI: 0.37 to 1.00, *P* = 0.05, I^2^ = 30% for heterogeneity, *P* = 0.23; Fig. 2a). In contrast, there was no significant difference in operation time between the two groups (WMD = − 11.68 d, 95% CI: − 34.10 to 10.75, *P* = 0.31, I^2^ = 87% for heterogeneity, *P* < 0.00001; Fig. 2b). However, the LS group showed less intraoperative blood loss than the OS group (WMD = − 115.27 ml, 95% CI: − 128.54 to − 101.99, *P* < 0.00001, I^2^ = 22% for heterogeneity, *P* = 0.25; Fig. 2c). Consistent with the results of the meta-analysis of intraoperative blood loss, the number of intraoperative blood transfusions was lower in the LS group than in the OS group (OR = 0.33, 95% CI: 0.21 to 0.52, *P* < 0.00001, I^2^ = 7% for heterogeneity, *P* = 0.38; Fig. 2d).

### Postoperative outcomes

**Fig. 3:**
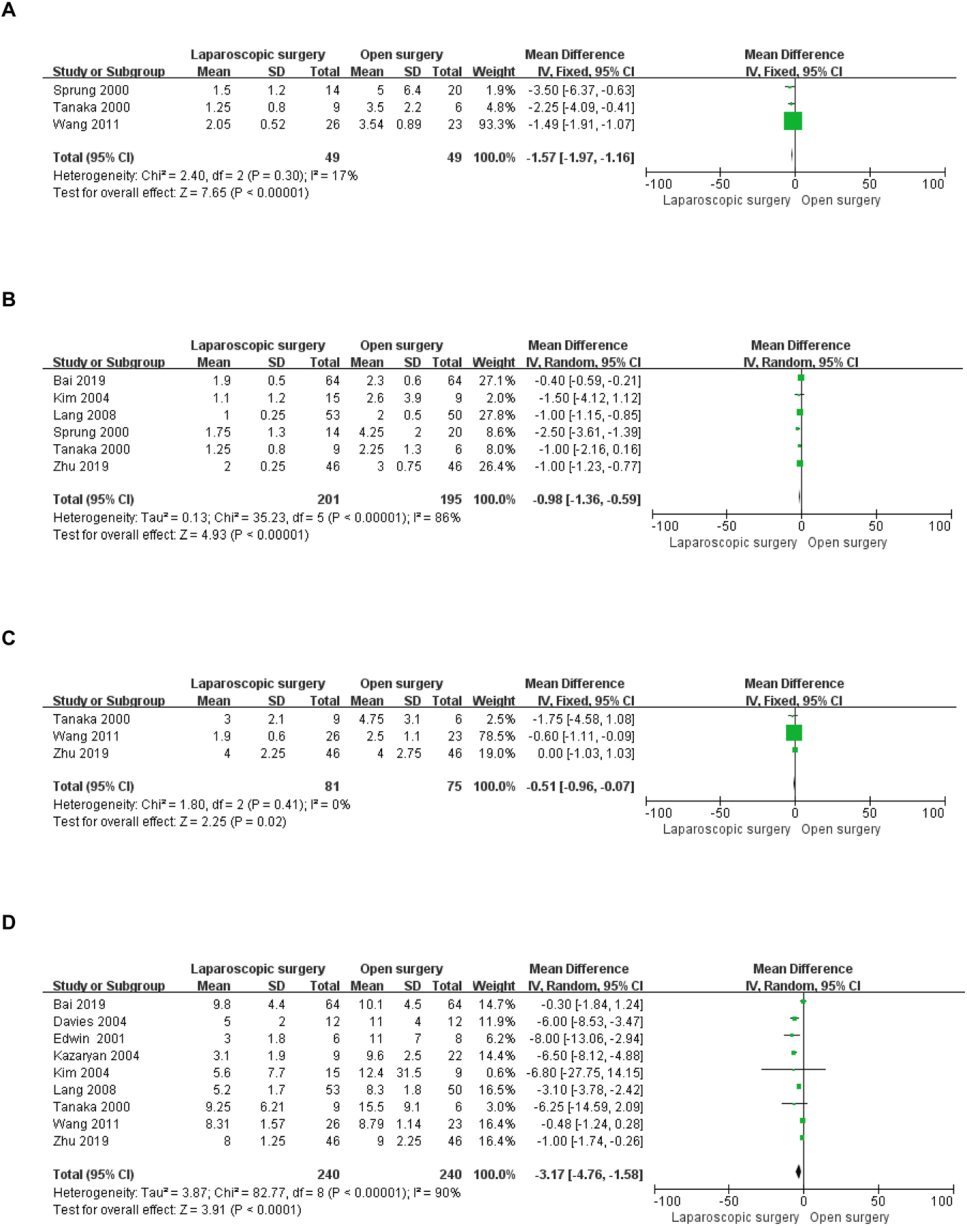
Forest plots of the meta-analysis for short-term outcomes. **a**: Time to ambulation was compared between the LS and OS groups. **b**: Time to oral intake was compared between the LS and OS groups. **c**: Length of drainage tube indwelling time was compared between the LS and OS groups. **d**: Length of postoperative hospital stay was compared between the LS and OS groups. LS: laparoscopic surgery; OS: open surgery; CI: confidence interval; SD: standard deviation

The postoperative outcome indicators included in the present study were as follows: time to ambulation and oral intake, drainage tube indwelling time, postoperative length of stay, complications, and blood pressure control. Data regarding time to ambulation after surgery were available in three included studies involving 98 patients [8, 25, 26]. Time to ambulation was shorter in the LS group than that in the OS group (WMD = − 1.57 d, 95% CI: − 1.97 to − 1.16, *P* < 0.00001, I^2^ = 17% for heterogeneity, *P* = 0.30; Fig. 3a). Time to resumption of eating was reported in six studies [13, 23–27], and the time to recovery of diet in the LS group was significantly shorter than that in the OS group (WMD = − 0.98 d, 95% CI: − 1.36 to − 0.59, *P* < 0.00001, I^2^ = 86% for heterogeneity, *P* < 0.00001; Fig. 3b). Three studies described the drainage tube indwelling time in 156 patients [8, 26, 27]; the drainage tube was removed earlier in the LS group than in the OS group after surgery (WMD = − 0.51 d, 95% CI: − 0.96 to − 0.07, *P* = 0.02, I^2^ = 0% for heterogeneity, *P* = 0.41; Fig. 3c). Nine studies including 480 patients presented postoperative hospital stays [8, 13, 19, 20, 22–24, 26, 27], and meta-analysis these data revealed shorter stays in the LS group than in the OS group (WMD = − 3.17 d, 95% CI: − 4.76 to − 1.58, *P* < 0.0001, I^2^ = 90% for heterogeneity, *P* < 0.00001; Fig. 3d).

**Fig. 4:**
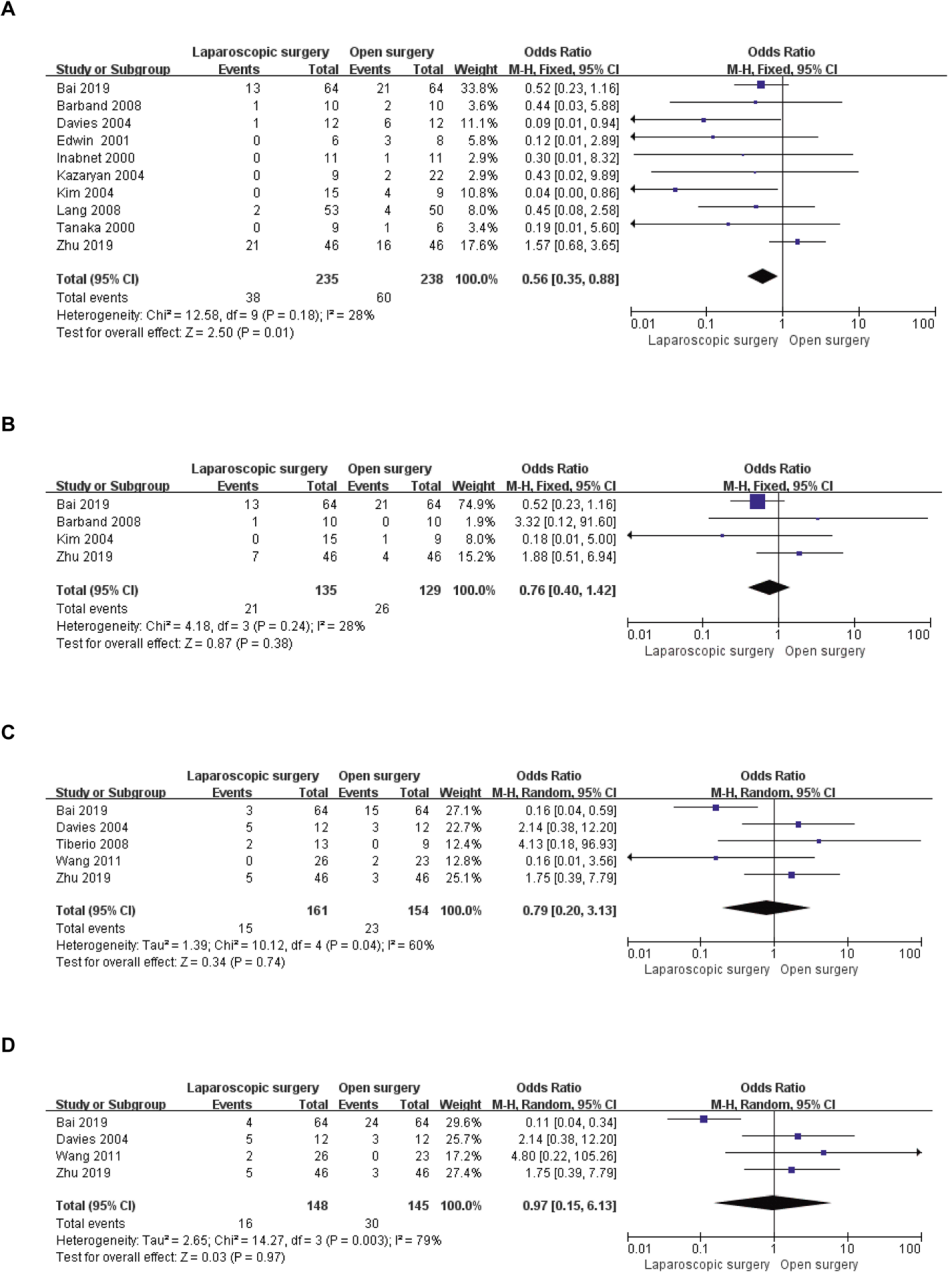
Forest plots of the meta-analysis for complications. **a**: Overall complications were compared between the LS and OS groups. **b**: Severe (Clavien-Dindo grade ≥ 2) complications were compared between the LS and OS groups. **c**: The number of postoperative hypotension cases was compared between the LS and OS groups. **d**: The number of postoperative CVDs was compared between the LS and OS groups. CVD: cardiovascular disease; LS: laparoscopic surgery; OS: open surgery; CI: confidence interval

Data for postoperative overall complications were available in ten of the included studies [13, 18–24, 26, 27], ranging from 0 to 45.6% in the LS group and from 8 to 50% in the OS group. Meta-analysis of these ten studies indicated that the likelihood of postoperative complications was lower in the LS group than in the OS group (OR = 0.56, 95% CI: 0.35 to 0.88, *P* = 0.01, I^2^ = 28% for heterogeneity, *P* = 0.18; Fig. 4a). The severity (Clavien-Dindo grade ≥ 2) of postoperative complications was included in four studies [13, 18, 23, 27], and no significant difference in severe postoperative complications between the two groups (OR = 0.76, 95% CI: 0.40 to 1.42, *P* = 0.38, I^2^ = 28% for heterogeneity, *P* = 0.24; Fig. 4b) was found. Five [7, 8, 13, 19, 27] and four [8, 13, 19, 27] studies reported postoperative hypotension and CVD, respectively. The rates of postoperative hypotension in the LS group were similar to those in the OS group (OR = 0.79, 95% CI: 0.20 to 3.13, *P* = 0.74, I^2^ = 60% for heterogeneity, *P* = 0.04; Fig. 4c), and no significant difference in CVD was detected (OR = 0.97, 95% CI: 0.15 to 6.13, *P* = 0.97, I^2^ = 79% for heterogeneity, *P* = 0.003; Fig. 4d).

**Fig. 5:**
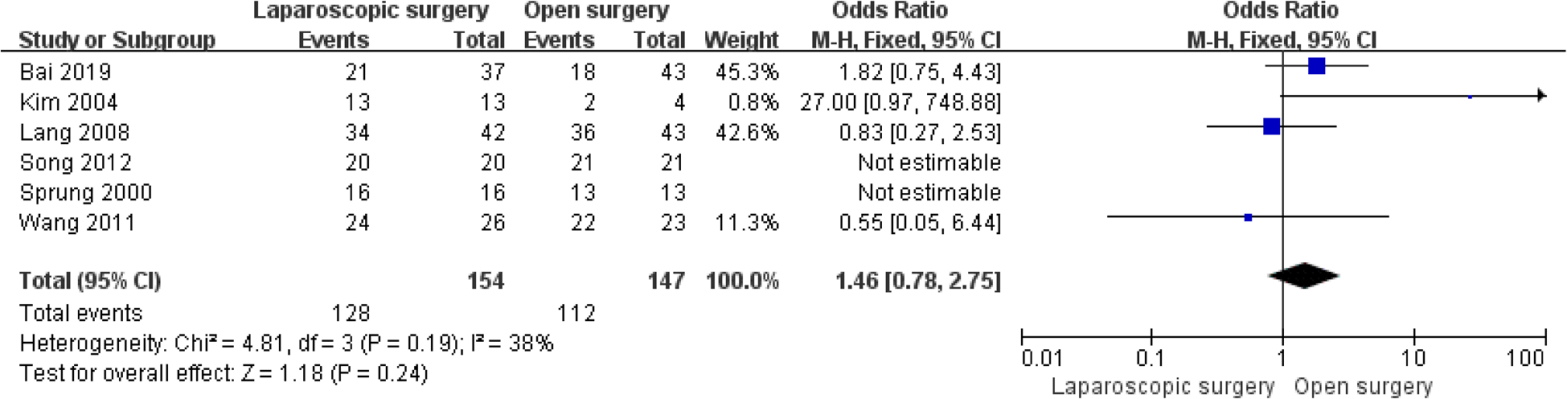
Forest plots of the meta-analysis of follow-up outcomes. The number of patients whose postoperative blood pressure returned to normal without drugs was compared between the LS and OS groups. LS: laparoscopic surgery; OS: open surgery; CI: confidence interval

In addition, data for postoperative blood pressure control during follow-up were available in six studies involving 301 patients [6, 8, 13, 23–25]. It should be noted that some patients had normal preoperative blood pressure and that all of the patients included in Wang’s study [8] were preoperatively diagnosed with hypertension. We compared the numbers of patients who were diagnosed with hypertension preoperatively and whose blood pressure was well controlled postoperatively without drugs for at least 3 months of follow-up. The results showed no statistically significant difference in the number of patients whose blood pressure returned to normal after surgery in either the LS group or the OS group (OR = 1.46, 95% CI: 0.78 to 2.75, *P* = 0.27, I^2^ = 38% for heterogeneity, *P* = 0.19; Fig. 5).

### Publication bias

**Fig. 6:**
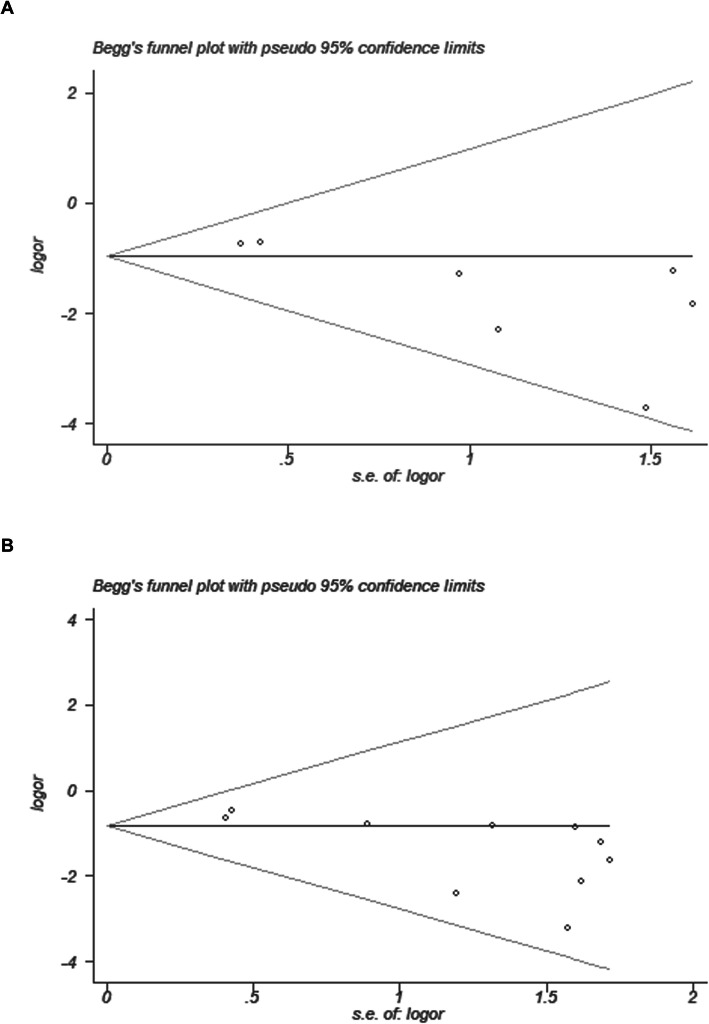
Funnel plots of the meta-analysis for numbers of intraoperative blood transfusions and postoperative complications. **a**: Intraoperative blood transfusions. **b**: Postoperative complications. RR: Relative risk

STATA software, version 12.0, was employed to generate funnel plots and Begg’s test for assessing publication bias of the number of intraoperative blood transfusions and overall postoperative complications in the two groups (Fig. 6). The funnel plot of the number of intraoperative blood transfusions displayed good symmetry, with all points falling within the 95% CI (Fig. 6a). Moreover, the *P* value of Begg’s test was greater than 0.05 (Begg’s test *P* = 0.133), suggesting no significant publication bias. According to the funnel plot of postoperative overall complications, as illustrated in Fig. 6b, points within the 95% CI accounted for almost all of them, and no significant publication bias in the pooled results was found (Begg’s test *P* = 0.592).

## Discussion

Surgery for PHEO is challenging because of the unpredictable release of catecholamine and rich blood supply. Prior to Gagner’s report on laparoscopic adrenalectomy for adrenal tumours [1], OS was the standard surgical approach for treating PHEO. However, laparoscopic device innovation and the accumulation of surgical experience have enabled performing laparoscopic resection for most PHEOs. Furthermore, an increasing number of robotic surgeries for PHEO have been reported with the development of this approach [28–30]. Nevertheless, the expensive purchase and maintenance costs for robots, the steep learning curve for robotic surgery in the treatment of PHEO and the lack of multi-centre prospective studies to confirm the safety and efficacy of the approach for PHEO have limited to some extent its application in the treatment of PHEO. Therefore, the most common surgical methods for the treatment of PHEO are still OS and LS. Several studies have compared the treatment outcomes of LS and OS for PHEO, but most had small sample sizes. We therefore performed this meta-analysis including 626 patients to reach a more compelling conclusion.

IHD is a major concern in the resection of PHEO. Preoperative use of alpha blockers and mild tumour manipulation are important to reduce the incidence of IHD [5, 31, 32]. With regard to IHD, pooled data revealed that LS provided more stable haemodynamics than OS (*P* = 0.05), which can be explained by the following factors. First, a magnified, clear laparoscopic field is helpful for the surgeon to perform fine dissection of the tumour. Second, direct manipulation of the tumour is reduced with the help of precise laparoscopic instruments. Third, in the magnified laparoscopic field, the adrenal main vein can be easily detected and ligated early, reducing the release of catecholamines into the blood. According to the Endocrine Society’s 2014 guidelines, open resection is recommended for PHEOs larger than 6 cm to ensure complete resection, reduce tumour rupture, and avoid local tumour recurrence [33]. In fact, there are few reports on laparoscopic resection of large PHEO [5, 13, 27, 31, 32, 34, 35]. An important note is that in all of the studies included in our meta-analysis, the majority of the tumours removed by LS were smaller than 6 cm and that all the results of this meta-analysis are based on this fact. Therefore, the difference in tumour size is a potential factor leading to the difference in haemodynamics between the two groups. No significant difference in operation time between the LS group and the OS group was found (*P* = 0.31). Different skills and experience among surgeons and the learning curve of surgery for PHEO might together contribute to the high heterogeneity of the pooled results in the comparison of operation times (I^2^ = 87%, *P* < 0.00001). Predictably, as laparoscopy matures, LS may require less time to remove PHEOs than that needed with OS. Haemorrhage during surgery is an important index for evaluating the quality of the operation [15], and a meaningful finding of this meta-analysis was that the LS group had less intraoperative blood loss than the OS group (*P* < 0.00001). Consistent with this, the rates of intraoperative blood transfusions were lower in the LS group than in the OS group (*P* < 0.00001). It is worth noting that the difference in tumour size between the LS group and OS group may influence the evaluation of outcomes, particularly in terms of intraoperative blood loss, transfusion rate and IHD, due to the need to increase tumour manipulation during the dissection and resection of large tumours.

Indicators of postoperative recovery, including time to ambulation, time to resuming eating, time to removing drainage, and postoperative hospital stays, were also evaluated in our meta-analysis. All of the indices in the laparoscopic group were superior to those in the open group, fully reflecting the minimally invasive advantages of LS.

Postoperative complications are key factors in determining the speed of postoperative recovery. Multiple comparisons of postoperative complications between the LS and OS groups were performed in this study. Our results showed a similar severe complication rate between the two groups (*P* = 0.38), though the rate of overall complications was lower in the laparoscopic group than in the open group (*P* = 0.01), suggesting that LS is a safe, effective and perhaps even superior surgical procedure for PHEO. After the resection of PHEO, the catecholamine level in the body is sharply reduced. In addition, with the continuous action of vasodilators used before or during the operation, patients are prone to postoperative hypotension or cardiovascular complications. Overall, there was no significant difference in the rates of postoperative hypotension (*P* = 0.74) and CVD (*P* = 0.97) between the two groups. Different postoperative management strategies conducted by different hospitals and doctors might have generated the heterogeneity of the aforementioned indicators of concern.

A good indicator of the effectiveness of surgical treatment for PHEO is the patient’s postoperative blood pressure control, which was assessed in this meta-analysis. However, the difference in the number of patients whose blood pressure returned to normal without drugs after surgery between the LS and OS groups was not statistically significant (*P* = 0.24).

Similar to other studies, our study had limitations. First, our meta-analysis compared the treatment outcomes of LS or OS for selective cases, such as unilateral, localized adrenal PHEO. Comprehensive studies including extra-adrenal, bilateral or malignant PHEOs should be performed to further perfect the results. Moreover, only one randomized, controlled trial (RCT) was included in this meta-analysis, and most of the studies were associated with a small number of enrolled patients and involved a single institution, suggesting that the quality of the evidence is not high. Given the above limitations, the pooled results in this meta-analysis should be considered with caution.

## Conclusions

The safety and efficacy of LS for PHEO are worthy of recognition. LS is associated with low rates of IHD, less intraoperative blood loss and lower transfusion rates and can provide a faster postoperative recovery than OS while achieving equal postoperative blood pressure control and no increased mortality compared to OS. More large randomized, controlled trials should be conducted to further confirm these advantages.

## Data Availability

All data generated or analysed during this study are included in this published article.

## Abbreviations

BMI: Body mass index
CI: Confidence interval
IHD: Intraoperative haemodynamic instability
LS: Laparoscopic surgery
OR: Odds ratio
OS: Open surgery
PHEO: Pheochromocytoma
WMD: Weight mean difference
